# Identifying At‐Risk Patients for Sustained Atrial High‐Rate Episodes Using the C_2_HEST Score: The West Birmingham Atrial Fibrillation Project

**DOI:** 10.1161/JAHA.120.017519

**Published:** 2021-03-05

**Authors:** Yan‐Guang Li, Daniele Pastori, Kazuo Miyazawa, Farhan Shahid, Gregory Y. H. Lip

**Affiliations:** ^1^ Liverpool Centre for Cardiovascular Science University of Liverpool and Liverpool Heart & Chest Hospital Liverpool United Kingdom; ^2^ Department of Cardiology and Institute of Vascular Medicine Peking University Third Hospital Beijing China; ^3^ Emergency Medicine Unit Department of Clinical Internal Anesthesiological and Cardiovascular Sciences Sapienza University Rome Italy; ^4^ Department of Clinical Medicine Aalborg University Aalborg Denmark

**Keywords:** atrial high rate episode, C_2_HEST score, risk evaluation, subclinical atrial fibrillation, Arrhythmias, Atrial Fibrillation

## Abstract

**Background:**

Sustained atrial high‐rate episodes (SAHREs) among individuals with a cardiac implantable electronic device are associated with an increased risk of adverse outcomes. Risk stratification for the development of SAHREs has never been investigated. We aimed to assess the performance of the C_2_HEST (coronary artery disease or chronic obstructive pulmonary disease [1 point each], hypertension [1 point], elderly [age ≥75 years, 2 points], systolic heart failure [2 points], thyroid disease [1 point]) score in predicting SAHREs in patients with cardiac implantable electronic devices without atrial fibrillation.

**Methods and Results:**

Five Hundred consecutive patients with cardiac implantable electronic devices in the West Birmingham Atrial Fibrillation Project in the United Kingdom were followed since the procedure to observe the development of SAHREs, defined by atrial high‐rate episodes lasting >24 hours. Risk factors and incidence of SAHREs were analyzed. The predictive value of the C_2_HEST score for SAHRE prediction was evaluated. Over a mean follow‐up of 53.1 months, 44 (8.8%) patients developed SAHREs. SAHREs were associated with higher all‐cause mortality (*P*<0.001) and ischemic stroke (*P*=0.001). Age and heart failure were associated with SAHRE occurrence. The incidence of SAHREs increased by the C_2_HEST score (39% higher risk per point increase). Among patients with a C_2_HEST score ≥4, the incidence of SAHREs was 3.62% per year (95% CI, 2.14–5.16). The C_2_HEST score had moderate predictive capability (area under the curve, 0.73; 95% CI, 0.64–0.81) and discriminative ability (log‐rank *P*=0.003), which was better than other clinical scores (CHA_2_DS_2_‐VASc, CHADS_2_, HATCH).

**Conclusions:**

The C_2_HEST score predicted SAHRE incidence in patients without atrial fibrillation who had an cardiac implantable electronic device, with the highest risk seen in patients with a C_2_HEST score ≥4 The benefit of using the C_2_HEST score in clinical practice in this patient population needs further investigation.

**Nonstandard Abbreviations and Acronyms**
AHRE: atrial high‐rate episode
C_2_HEST: coronary artery disease or chronic obstructive pulmonary disease (1 point each), hypertension (1 point), elderly (age ≥75 years, 2 points), systolic heart failure (2 points), thyroid disease (1 point)
CHADS_2_: congestive heart failure, hypertension, age ≥75 years, diabetes mellitus, stroke or transient ischemic attack
CHA_2_DS_2_‐VASc: congestive heart failure, hypertension, age ≥75 years, diabetes mellitus, stroke or transient ischemic attack, vascular disease, age 65 to 74 years, sex category
HATCH: hypertension, age ≥75 years, transient ischemic attack or stroke, chronic obstructive pulmonary disease, and heart failure
OAC: oral anticoagulation
SAHRE: sustained atrial high‐rate episode

**Clinical Perspective**

**What Is New?**

- Sustained atrial high‐rate episodes in patients with cardiac implantable electronic device are common, with age and heart failure being independent risk factors.
- The risk of sustained atrial high‐rate episodes can be stratified by the C_2_HEST (coronary artery disease or chronic obstructive pulmonary disease [1 point each], hypertension [1 point], elderly [age ≥75 years, 2 points], systolic heart failure [2 points], thyroid disease [1 point]) score with moderate predictive accuracy.

**What Are the Clinical Implications?**

- For high‐risk patients (C_2_HEST score ≥4), a more intensive (eg, shorter follow‐up interval) or continuous (eg, remote monitoring) device interview may be needed, enabling early detection and early treatment of subclinical atrial fibrillation.

Atrial high‐rate episodes (AHREs) are common among patients with cardiac implantable electronic devices (CIEDs), with a prevalence ranging from 30%^1^ to 70%.^2^ In patients without atrial fibrillation (AF), the prevalence of AHREs is ≈30%.^3^, ^4^ Patients with AHREs are at significantly increased risk of developing overt AF, and, for this reason, AHREs are regarded to as “subclinical AF.” Indeed, AHRE represents a significant clinical entity, with the increasing application of CIED and its detection of infrequent atrial arrhythmias through continuous long‐term heart rhythm monitoring.

The occurrence of an AHRE has been associated with an adverse clinical prognosis,^2^ including increased thromboembolism (hazard ratio [HR], 3.40; 95% CI, 1.38–8.37), mortality (HR, 3.47; 95% CI, 1.51–7.95),^5^, ^6^ and heart failure (HF) (HR, 4.58; 95% CI, 1.64–12.8).^7^ The minimum duration of AHRE conferring increased thromboembolic risk is not well defined, but there is a growing evidence showing that sustained AHRE (SAHRE) lasting >24 hours is associated with an increased risk of thromboembolic events.^5^, ^8^, ^9^ In a subanalysis from the ASSERT (Asymptomatic Atrial Fibrillation and Stroke Evaluation in Pacemaker Patients and the Atrial Fibrillation Reduction Atrial Pacing Trial),^5^ SAHRE was associated with significantly increased risk of subsequent stroke or systemic embolism (HR, 3.24; 95% CI, 1.51–6.95), while no significant risk difference was observed between patients with AHRE of 6 minutes to 24 hours or those without AHRE.^5^ Similarly, Capucci et al^8^ reported that only AF episodes lasting >24 hours were independently related with embolism (odds ratio, 3.1; 95% CI, 1.1–10.5). Cori et al^9^ also showed that AF/AHREs lasting >24 hours confers a critical higher thromboembolic risk (>4.1% per year) compared with those AF/AHREs lasting <24 hours (<1.68% per year). These results show that there may be a dose‐effect relationship between AHRE and thromboembolism, implying that SAHRE warrants more clinical considerations.

For these reasons, a consensus document from the European Heart Rhythm Association^10^ warranted an early detection of patients with SAHRE through careful examination of intracardiac electrograms. This may allow prompt detection of AF and timely initiation of anticoagulation, thus reducing the risk of stroke. In a retrospective study including 4388 patients with new‐onset AF, 98 strokes (2.2%) occurred between AF diagnosis and oral anticoagulation (OAC) initiation.^11^ Thus, early detection of AF and early initiation of anticoagulation is crucial for avoiding strokes. In a retrospective study including 71 CIEDs patients with previous transient ischemic attack (TIA)/stroke, intensive remote monitoring was associated with reduced risks of all‐cause mortality (2.2% per year versus 8.1% per year) and hospitalization (5.8% per year versus 9.7% per year).^12^

There is still a lack of evidence on which group of patients with CIEDs may benefit from more intensive device evaluation or continuous remote monitoring regarding AF detection. We have previously demonstrated that some clinical conditions and laboratory biomarkers are associated with the development of AHRE.^13^ However, no risk evaluation model for the development of SAHRE has been developed.

Recently, a new clinical risk evaluation, the C_2_HEST (coronary artery disease or chronic obstructive pulmonary disease [1 point each], hypertension [1 point], elderly [age ≥75 years, 2 points], systolic HF [2 points], thyroid disease [1 point]) score, has been developed and validated to predict incident AF.^14^ The C_2_HEST score has been validated in diverse populations with modest to moderate predictive capability.^15^, ^16^, ^17^, ^18^, ^19^ SAHRE has been recognized as “subclinical AF” and shares many common characteristics and risk factors with clinical AF. In this study, we investigated the hypothesis of whether the C_2_HEST score was suitable in assessing the risk of developing SAHRE in patients with a CIED and no history of AF.

## Methods

The data that support the findings of this study are available from the corresponding author upon reasonable request. The data set of patients with CIEDs, which belongs to the West Birmingham Atrial Fibrillation Project, has been previously described.^6^, ^13^ Consecutive patients who have attended the cardiology department of Sandwell and West Birmingham Hospitals between January 1999 to January 2017 were retrospectively reviewed. All patients with a permanent pacemaker, implantable cardioverter‐defibrillator, or cardiac resynchronization therapy device were followed since the implantation procedure. Patients’ information was collected from the Clinical Data Archive of the National Health System. Patients with known AF at baseline were excluded from the analysis, considering it has a significant impact on the incidence of SAHRE. The ascertainment of previous AF was majorly based on medical history. For patients receiving anticoagulation and antiarrhythmics at baseline, the indications for such medications were also checked to ensure that they were not being prescribed for known previous AF. Conditions regarding AF presence and laboratory and echocardiography data were limited within 1 year before patient inclusion of the current study.

AHRE was defined as an atrial rate ≥175 beats per minute, and SAHRE was defined as a single AHRE lasting >24 hours. The identification and duration of AHRE events were automatically recorded by each device. The records of AHRE events were reviewed by arrhythmia specialists in charge of device follow‐up and uploaded to local websites of an individual device company. Baseline demographic characteristics, medical histories, medications, echocardiography, and laboratory test results were entered into a uniformed data framework, which was conducted by 3 independent investigators (D.P., K.M., and Y.G.L). Data collection and secondary verification were also performed to guarantee quality and accuracy. C_2_HEST; CHA_2_DS_2_‐VASc (congestive HF, hypertension, age ≥75 years, diabetes mellitus, stroke or TIA, vascular disease, age 65 to 74 years, sex category); CHADS_2_ (congestive HF, hypertension, age ≥75 years, diabetes mellitus, stroke or transient ischemic attack); and HATCH (hypertension, age ≥75 years, TIA or stroke, chronic obstructive pulmonary disease, and HF) scores were calculated according to their original descriptions.^14^, ^20^, ^21^

This study was conducted in accordance with the European Union Guidance on Good Clinical Practice CPMP/ECH/135/95 and the Declaration of Helsinki. Patients were not involved in data extraction, and there was no impact on their medical/social care. Ethical review was therefore not required with our use of anonymized data, and patient consent was not sought.

### Statistical Analysis

Continuous and categorical variables were expressed as mean±SD, median (interquartile range), or observed numbers (percentages), wherever appropriate. Differences between groups were tested using Student *t* test (normally distributed variable), Mann‐Whitney *U* test (non‐normal distributed data), and chi‐square test (categorical variable). Patients were grouped according to C_2_HEST score as per the original article (low risk: 0 or 1 point, medium risk: 2 or 3 points, high risk: ≥4 points). The incident rate and 95% CIs of patients with and without SAHRE ≥24 hours were calculated. Kaplan‐Meier curves were used to depict the accumulative hazard of patients developing SAHRE. For depicting Kaplan‐Meier curves, only the first recorded SAHRE at follow‐up was taken into account for the outcome event. The difference between Kaplan‐Meier curves among the 3 groups was analyzed using log‐rank test. The HRs and 95% CIs of patients with different risk categories were assessed using a Cox proportional hazard model. Potential predictive factors of SAHRE in the univariable analysis with *P*<0.1 were included in the multivariable model. For assessment of the discriminative performance of the C_2_HEST score in predicting SAHRE, receiver operating characteristic curve analysis and Delong test were performed.

We built 2 different survival models: model 1, which included all significant risk factors at multivariable Cox proportional hazards regression analysis; and model 2, which included the same factors of model 1 plus the C_2_HEST score. The performances of the 2 models were then compared. Goodness‐of‐fit test was conducted using Hosmer‐Lemeshow test, which describes the difference between expected (by score) and observed number of events.

Given the underlying influence of antiarrhythmic drugs on SAHRE, sensitivity analysis with receiver operating characteristic curves and Kaplan‐Meier curves (log‐rank test) were conducted among individuals not taking antiarrhythmic drugs (amiodarone, β‐blockers, and digoxin). We also tested the performance of the C_2_HEST score in predicting short‐term AHRE (≥5 minutes). A 2‐tailed *P* value <0.05 was considered statistically significant. Analyses were performed using SPSS Statistics version 23.0 (IBM), STATA software version 15.0 (StataCorp LLC), and MedCalc software version 18.11 (MedCalc Software Ltd).

## Results

The baseline characteristics of the studied population are shown in Table 1. After excluding patients with known AF at baseline, 500 patients with CIEDs were included in the present study. A permanent pacemaker was the most frequently used device (94.3%), followed by implantable cardioverter‐defibrillator (4.4%) and cardiac resynchronization therapy (1.2%). The most prevalent reasons for device implantation were atrioventricular block (56.2%) and sick sinus syndrome (24.8%). The mean age of the population was 69.9 years, of which 57.0% were men. Hypertension (70.2%), hyperlipidemia (58.6%), and diabetes mellitus (27.0%) were the most prevalent comorbidities. For patients receiving anticoagulation, the indications included previous pulmonary embolism, deep venous thrombosis, and hip replacement (n=34 in total). In addition, 87 patients (78 in the SAHRE group and 9 in the non‐SAHRE group) were taking β‐blockers for hypertension, HF, or coronary artery disease rather than arrhythmias. Six patients were taking digoxin for HF, and 17 were taking amiodarone for ventricular arrhythmias. In the 17 patients who were treated with amiodarone, 9 were implanted with an implantable cardioverter‐defibrillator, 1 with a cardiac resynchronization therapy device, and 7 had permanent pacemakers. Among patients with HF, HF with reduced ejection fraction was the predominant type and only 2 patients had HF with preserved ejection fraction, which was recognized as those with symptoms or physical signs of HF and with left ventricular ejection fraction >50% on echocardiography.

**Table 1:** Baseline Characteristics of the Patients

Characteristics	Total (N=500)	No SAHRE (n=456)	SAHRE (n=44)	*P* Value
Men, n (%)	285 (57.0)	252 (55.3)	33 (75.0)	0.012
Age, mean±SD	69.9±14.6	69.3±14.9	75.6±9.3	<0.001
Age ≥65 y, n (%)	356 (71.2)	315 (69.1)	41 (93.2)	0.001
Age ≥75 y, n (%)	233 (46.6)	205 (45.0)	28 (63.6)	0.018
Comorbidities, n (%)				
Hypertension	351 (70.2)	316 (69.3)	35 (79.5)	0.156
DM	135 (27.0)	120 (26.3)	15 (34.1)	0.268
Hyperlipidemia	293 (58.6)	261 (57.2)	32 (72.7)	0.145
CAD	134 (26.8)	117 (25.7)	17 (38.6)	0.064
Stroke/TIA	50 (10.0)	44 (9.6)	6 (13.6)	0.400
COPD	22 (4.4)	20 (4.4)	2 (4.5)	0.961
HF	15 (3.0)	11 (2.4)	4 (9.1)	0.013
Hyperthyroidism	6 (1.2)	4 (0.9)	2 (4.5)	0.033
Device type, n (%)				
PPM	472 (94.4)	431 (94.5)	41 (93.2)	0.713
ICD	22 (4.4)	19 (4.2)	3 (6.9)	0.413
CRT	6 (1.2)	6 (1.3)	0 (0.0)	0.444
Indications for device implantation, n (%)				
Sick sinus syndrome	124 (24.8)	115 (25.2)	9 (20.5)	0.545
Atrioventricular block	281 (56.2)	254 (55.7)	27 (61.4)	0.908
Syncope	43 (8.6)	41 (9.0)	2 (4.5)	0.337
HF	4 (0.8)	4 (0.9)	0 (0.0)	0.534
VT/VF	22 (4.4)	19 (4.2)	3 (6.8)	0.423
Laboratory results				
eGFR, mL/min per 1.73 m^2^	69.4±22.4	69.8±22.3	65.6±23.7	0.201
Creatinine, µmol/L	94.3±39.5	93.6±39.8	101.7±35.8	0.240
White blood cell count (×10^9^/L)	7.90±5.01	7.75±2.40	9.52±15.1	<0.001
Echocardiography				
LAD, mm	41.1±8.7	40.5±8.4	45.9±10.4	0.021
LAV, mL	54.4±23.5	52.2±20.6	71.0±35.3	0.011
LAVI, mL/m^2^	30.3±13.0	29.1±11.0	39.5±21.0	0.015
LVEF (%)	57.4±14.0	58.0±13.8	52.9±15.4	0.076
Medication, n (%)				
β‐Blocker	87 (17.4)	78 (17.1)	9 (20.5)	0.852
Digoxin	6 (1.2)	5 (1.1)	1 (2.3)	0.567
Amiodarone	17 (3.4)	15 (3.3)	2 (4.5)	0.777
Diuretic	114 (22.8)	102 (22.4)	12 (27.3)	0.743
Statin	270 (62.5)	237 (52.0)	33 (75.0)	0.024
CCB	124 (24.8)	113 (24.8)	11 (25.0)	0.698
Anticoagulant	34 (6.8)	27 (5.9)	7 (16.0)	0.030
Antiplatelet	224 (44.8)	202 (44.3)	22 (50.0)	0.942
ACEI/ARB	212 (42.4)	185 (40.6)	27 (61.4)	0.040
CHA_2_DS_2_‐VASc	3.1±1.7	3.0±1.7	3.7±1.5	0.009

ACEI indicates angiotensin‐converting enzyme inhibitor; ARB, angiotensin receptor blocker; CAD, coronary artery disease; CCB, calcium channel blocker; CHA_2_DS_2_‐VASc, congestive heart failure, hypertension, age ≥75 years, diabetes mellitus, stroke or transient ischemic attack, vascular disease, age 65 to 74 years, sex category; COPD, chronic obstructive pulmonary disease; CRT, cardiac resynchronization therapy; DM, diabetes mellitus; eGFR, estimated glomerular filtration rate; HF, heart failure; ICD, implantable cardioverter‐defibrillator; LAD, left atrium diameter; LAV, left atrial volume; LAVI, left atrial volume index; LVEF, left ventricular ejection fraction; PPM, permanent pacemaker; SAHRE, sustained atrial high‐rate episode; TIA, transient ischemic attack; VF, ventricular fibrillation; and VT, ventricular tachycardia.

### SAHRE Incidence and Risk Factors

During a mean follow‐up of 53.1 months (SD=33.9) (SAHRE group: 51.8 months [SD=34.0]; non‐SAHRE group: 66.7 months [SD=29.7]), 168 patients had an AHRE (>5 minutes), among which 44 developed SAHREs (>24 hours), with an incident rate of 2.09% per year (95% CI, 1.47%–2.71% per year). Patients with SAHREs were more frequently men, older, and more likely to have hyperthyroidism and HF (*P*<0.05, respectively). Larger left atrium diameter, left atrium volume, and left atrium volume index were observed in patients with SAHRE (*P*<0.05, respectively). Patients with SAHREs had significantly higher CHA_2_DS_2_‐VASc scores, with 95.5% (n=42) of patients having a CHA_2_DS_2_‐VASc score ≥2 (men) or 3 (women) compared with 78.3% (n=357) patients without SAHREs (*P*=0.007) (Table 1).

In univariable Cox proportional hazard regression analysis, male sex, age ≥75 years, HF, and white blood cell counts were associated with SAHREs (Table 2). In adjusted modeling, male sex, age ≥75 years, HF, and white blood cell count were independently associated with SAHREs (Table 2). In the multivariable model analysis, age ≥75 years and HF were the 2 strongest risk factors (Table 2).

**Table 2:** Risk Factors Associated With the Development of SAHRE

Characteristics	Univariable Analysis			Multivariable Analysis			Univariable AUC	Adjusted AUC
	HR	95% CI	*P* Value	HR	95% CI	*P* Value		
Men	2.14	1.08–4.23	0.029	0.53	1.04–4.17	0.039	0.60	0.53
Age ≥75 y	2.93	1.57–5.45	0.001	0.55	1.36–4.84	0.004	0.66	0.55
Hyperthyroidism	1.00	0.53–1.89	0.998					
COPD	0.82	0.20–3.39	0.783					
Hypertension	1.67	0.80–3.47	0.170					
DM	1.65	0.88–3.08	0.117					
HF	4.02	1.43–11.26	0.008	3.02	1.06–8.57	0.038	0.54	0.63
Stroke/TIA	1.87	0.79–4.46	0.156					
Vascular disease	1.55	0.85–2.83	0.158					
CAD	1.46	0.79–2.69	0.223					
WBC	1.04	1.02–1.07	0.001	1.04	1.01–1.06	0.005	0.51	0.51
LAD	1.33	0.85–2.09	0.209					
eGFR	0.99	0.98–1.00	0.166					

AUC indicates area under the curve; CAD, coronary artery disease; COPD, chronic obstructive pulmonary disease; DM, diabetes mellitus; eGFR, estimated glomerular filtration rate; HF, heart failure; HR, hazard ratio; LAD, left atrial dimension; SAHRE, sustained atrial high‐rate episode; TIA, transient ischemic attack; and WBC, white blood cell.

### C_2_HEST Score and Risk of SAHRE

The mean C_2_HEST score was 2.5±1.6. Patients with SAHREs had a significantly higher mean C_2_HEST score compared with those without SAHREs (3.4±1.5 versus 2.4±1.6, respectively; *P*<0.001). Patients were categorized into 3 groups according to C_2_HEST score risk, ie, low‐risk: 0 or 1 point (28.8%, n=144), medium risk: 2 or 3 points (42.2%, n=211), and high risk: ≥4 points (29.0%, n=145) (Table 3). The follow‐up durations were 50.9±32.5 months, 53.9±37.2 months, and 54.1±30.3 months for the low‐, medium‐, and high‐risk groups, respectively. The risk of SAHREs increased by the C_2_HEST score (Table 3). Using patients with C_2_HEST score 0 or 1 as the reference group, the HR for the risk of SAHREs increased in the medium‐ and high‐risk groups (Table 3). For each point increase of the C_2_HEST score, there was a 39% higher risk of developing SAHREs (HR, 1.39; 95% CI, 1.16–1.68).

**Table 3:** Risk of SAHRE According to the C_2_HEST Score Groups

C_2_HEST Score	Patients, n (%)	SAHRE, n	Incident Rate* (95% CI)	HR (95% CI)	*P* Value
0 or 1	144 (28.5)	5	0.85 (0.27–1.94)	Reference	Reference
2 or 3	211 (42.2)	17	1.87 (1.06–2.91)	2.06 (0.76–5.59)	0.159
≥4	145 (29.0)	22	3.62 (2.14–5.16)	4.25 (1.61–11.22)	0.004

C_2_HEST indicates coronary artery disease or chronic obstructive pulmonary disease (1 point each), hypertension (1 point), elderly (age ≥75 years, 2 points), systolic heart failure (2 points), thyroid disease (1 point); HR, hazard ratio; and SAHRE, sustained atrial high‐rate episode.

* Per 100 patient‐years (crude rate).

**Figure 1:**
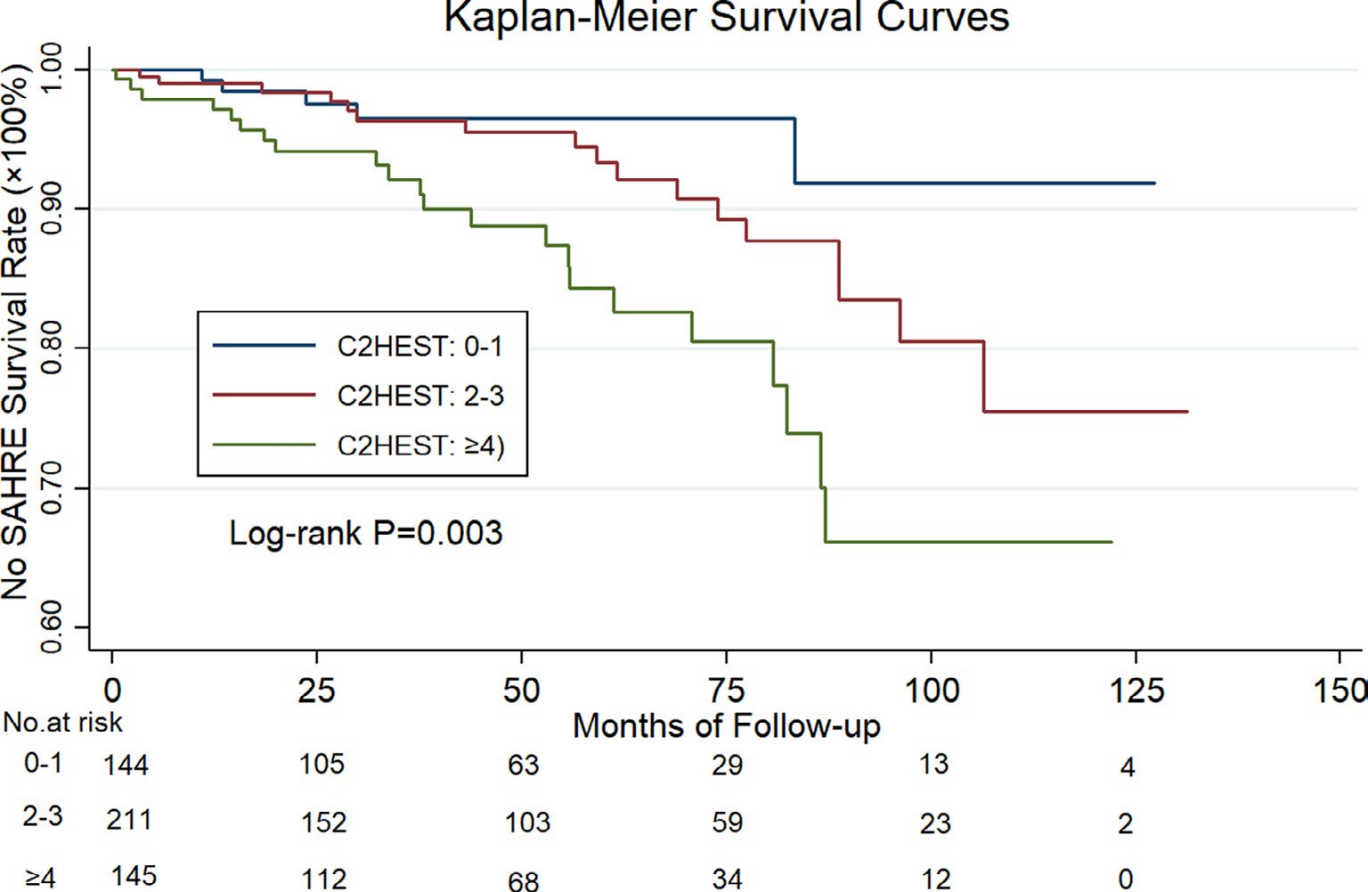
The cumulative survival rates of sustained atrial high‐rate episodes (SAHREs) with regard to the C_2_HEST (coronary artery disease or chronic obstructive pulmonary disease [1 point each], hypertension [1 point], elderly [age ≥75 years, 2 points], systolic heart failure [2 points], thyroid disease [1 point]) score. Kaplan‐Meier curves depict the different accumulative survival rates free from SAHRE regarding C_2_HEST score risk groups. A significant higher hazard is seen among the high‐risk subgroup compared with the low‐ and medium‐risk groups (log‐rank *P*=0.003).

As depicted by the survival curve, patients with a C_2_HEST score ≥4 showed a higher risk for SAHRE development compared with those in the low‐ and medium‐risk groups (log‐rank test, *P*=0.003) (Figure 1). No difference was found between the low‐ and medium‐risk groups (*P*=0.159). When we divided patients into 2 groups, such as high risk (C_2_HEST ≥4) and low‐medium risk (C_2_HEST score 0–3), we found a significant difference in the incidence of SAHRE (IR 3.62% per year versus 1.47% per year; log‐rank, *P*=0.001).

The C_2_HEST score showed moderate predicting accuracy for SAHREs with an area under the curve (AUC) of 0.73 (95% CI, 0.64–0.81). The test for goodness‐of‐fit was not significant (*P*=0.931).

Model 1 (including sex, age, HF, and white blood cell count) had an AUC of 0.66 (95% CI, 0.57–0.69) and model 2 (including sex, age, HF, white blood cell count, and C_2_HEST) showed an AUC of 0.74 (95% CI, 0.64–0.79), which was superior to model 1 (*P*=0.033).

### Secondary Analyses

Among patients with a history of ischemic stroke/TIA, SAHRE showed better predictive capability (AUC=0.77; 95% CI, 0.58–0.95). Among patients with a permanent pacemaker, the AUC for the C_2_HEST score was 0.70 (95% CI, 0.62–0.77).

**Figure 2:**
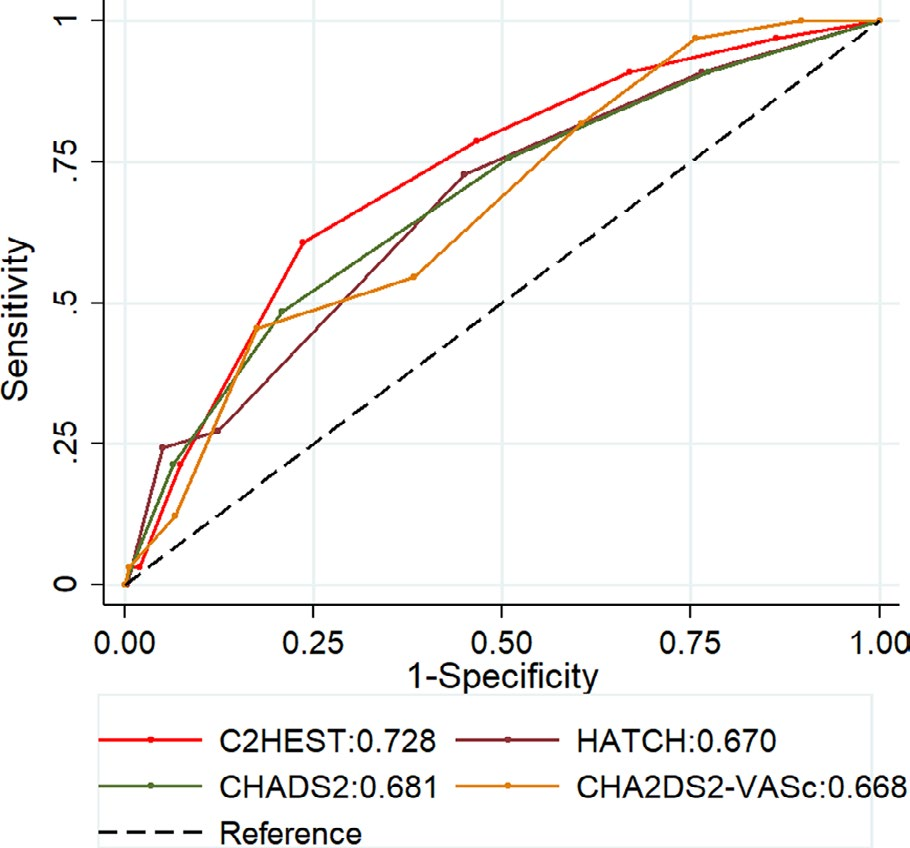
Performance of the risk scores for predicting sustained atrial high‐rate episodes. C2HEST indicates coronary artery disease or chronic obstructive pulmonary disease (1 point each), hypertension (1 point), elderly (age ≥75 years, 2 points), systolic heart failure (2 points), thyroid disease (1 point); CHADS2, congestive heart failure, hypertension, age ≥75 years, diabetes mellitus, stroke or transient ischemic attack; CHA2DS2‐VASc, congestive heart failure, hypertension, age ≥75 years, diabetes mellitus, stroke or transient ischemic attack, vascular disease, age 65 to 74 years, sex category; and HATCH, hypertension, age ≥75 years, transient ischemic attack or stroke, chronic obstructive pulmonary disease, and heart failure.

We compared the C_2_HEST score with other scoring systems such as CHA_2_DS_2_‐VASc, CHADS_2_, and the HATCH. We found that the C_2_HEST score showed a better predictive of SAHRE (versus CHA_2_DS_2_‐VASc 0.67 [0.58–0.76], *P*=0.128; CHADS_2_ 0.68 [0.58–0.78], *P*=0.307; and HATCH 0.67 [0.58–0.76], *P*=0.089) (Figure 2).

We also tested the risk stratification capacity of the C_2_HEST score for the AHREs ≥5 minutes. The prevalence of AHREs ≥5 minutes increased with C_2_HEST risk groups (24.3%, 31.8%, and 45.5% for the low‐, medium–, and high‐risk groups, respectively; log‐rank *P*=0.001, figure not shown). The risk of AHRE increased by 23.8% for each 1‐point increase in the C_2_HEST score.

### SAHRE and Clinical Outcomes

During follow‐up, thromboembolic events were found in 27 (5.4%) patients, 30 (6.0%) experienced acute HF, 10 (2.0%) were hospitalized for cardiovascular reasons, and 28 (5.8%) died. Major bleeding was seen in 9 (1.8%) patients and minor bleeding in 25 (5.5%) patients.

Patients who developed SAHREs had significantly higher risks of all‐cause death (4.1% per year versus 1.0% per year, *P*<0.001) and ischemic stroke (5.3% per year versus 4.2% per year, *P*=0.001). Patients with SAHREs were older and were more likely male and with HF. No significant differences were seen between patients with and without SAHREs regarding myocardial infarction, acute HF, bleeding, and cardiovascular hospitalization (*P*>0.05, respectively).

Among the 44 patients who developed SAHREs at follow‐up, 7 patients were taking OAC at baseline, because of previous embolic events, including pulmonary embolism or deep vein thrombosis; and 23 patients were prescribed an OAC agent after the development of AHRE. The mean time interval between the date of AHRE development to OAC prescription was 573 days, implying a probable delayed treatment of subclinical AF. Among the 23 patients, 3 were started with an OAC agent after embolic events (2 ischemic strokes and 1 systemic embolism).

## Discussion

Our study suggests that the C_2_HEST score has moderate predictive accuracy for SAHRE in patients implanted with a CIED and without a history of AF. The risk of SAHRE gradually increased by the C_2_HEST score. Furthermore, we confirm that SAHRE was associated with worse clinical outcomes, including all‐cause mortality and ischemic stroke.

Only a few studies have focused on the risk factors for AHREs lasting >5 or 6 minutes.^22^, ^23^ However, SAHREs lasting >24 hours are more likely to represent a “subclinical” AF condition, needing proactive management given its association with thromboembolic events and mortality. In the present study, we found that age and HF were the most significant risk factors for SAHREs, which is similar to that of the risk factors for incident AF.^14^, ^18^, ^24^

In the ASSERT trial, 10.7% of patients developed SAHREs during 2.5 years of follow‐up,^5^ with an estimated incidence of ≈4.3% per year. All of those enrolled patients were older than 65 years with hypertension and an enlarged left atrium at baseline. In the present study, we found a prevalence of SAHRE of 8.8% with an incident rate of 2.09% per year, which is lower than in the ASSERT study, probably related to a relatively younger population (70 versus 76 years).

To identify patients at risk of SAHREs we used the C_2_HEST score, primarily designed for incident AF.^14^ The C_2_HEST score showed moderate ability in assessing the individual risk for SAHRE development (AUC=0.728), which was consistent in other critical subgroups of patients, such as those with a history of ischemic stroke/TIA. The incident rate of SAHRE increased by the C_2_HEST score, with a 39% higher risk for SAHRE for each point increase of the score. In particular, among patients with a C_2_HEST score ≥4, nearly 1 in 6 developed SAHREs during follow‐up, which may enable clinicians to flag those patients who warrant more intense device review or continuous remote monitoring. Close monitoring (eg, ≤3 months) of patients with a C_2_HEST score ≥4 would help in identifying patients who develop SAHREs earlier than “routine” follow‐up, eg 6 or 12 months. Furthermore, remote monitoring could be more effective in identifying SAHREs, as shown by the CRYSTAL AF (Cryptogenic Stroke and Underlying AF) study, in which remote monitoring identified significantly more patients with AHREs within 6 months compared with standard follow‐up (8.9% versus 1.4%; HR, 6.4 [95% CI, 1.9–21.7]).^25^ Indeed, compared with standard scheduled device follow‐up, remote monitoring could detect AF/AHRE 1 to 5 months earlier.^26^ Concerning cost‐effectiveness, remote monitoring might even be more precisely applied to at‐risk patients for SAHRE (C_2_HEST score ≥4), waiving the need to monitor non–high‐risk patient groups, which needs further investigation.

Nevertheless, many issues regarding AHRE are not definitive, including lack of a temporal relationship between AHREs and thromboembolic events.^27^ Current evidence in the literature does not support routine anticoagulation of SAHREs if there is no clinical overt AF being diagnosed, given that we still do not know whether the embolic risk could be mitigated or prevented by anticoagulation, and whether the increased risk of bleeding is acceptable. The efficacy and safety of OAC for stroke prevention in AHREs is under investigation (the ARTESiA [Apixaban for the Reduction of Thrombo‐Embolism in Patients With Device‐Detected Sub‐Clinical Atrial Fibrillation]^28^ and NOAH‐AFNET 6 [Non–vitamin K Antagonist Oral Anticoagulants in Patients With Atrial High Rate Episodes]^29^ trials).

We also compared the C_2_HEST score with other clinical risk scores such as the CHADS_2_, CHA_2_DS_2_‐VASc, and HATCH scores. The predictive value of the C_2_HEST score was similar to CHADS_2_ and CHA_2_DS_2_‐VASc and slightly better than the HATCH score. However, these scores were proposed to predict stroke in AF (CHADS_2_ and CHA_2_DS_2_‐VASc) and AF progression (HATCH), and not for predicting incident AF or AHRE. A recent EHRA consensus document appealed for using the appropriate risk score for what they were proposed for, and not to use scores to predict outcomes for which they were not designed.^30^

In the present study, we found that SAHRE was associated with a significantly increased risk of all‐cause mortality and stroke, which is similar to a recent study showing that patients with SAHREs had a higher risk of mortality (HR, 2.67).^31^ Previously, we have shown that AHRE was related to higher major adverse cardiovascular events.^32^ Altogether these findings suggest that the occurrence of AHREs may worsen the prognosis of patients.

### Strengths and Limitations

We have demonstrated that SAHRE is not uncommon among patients with CIEDs. A high‐risk subgroup of patients for SAHRE could be identified through a scoring system, namely the C_2_HEST score. This study also represents the first application of the C_2_HEST score for predicting SAHRE. The C_2_HEST score is a user‐friendly score that may allow better risk stratification in this patient population.

Nevertheless, there are some limitations in the present study. This is a retrospective study with a relatively limited sample size, claiming for a validation in larger population studies. Although a single episode of AHRE lasting >24 hours was defined as an SAHRE, there is a certain variability for its definition. Thus, an atrial rate ranging from 170 beats per minute to 200 beats per minute has been used in previous studies. We have used the most frequently used and reliable criteria (from the device programming aspect) to define AHRE, which may have a slight difference compared with other cutoff values. The performance of the C_2_HEST score may vary in populations with a different incidence of chronic obstructive pulmonary disease and thyroid diseases. Of note, these 2 conditions may be underdiagnosed in clinical practice; our study suggests that physicians managing patients with CIEDs should make efforts in detecting these 2 comorbidities to better stratify the risk of SAHREs. Although the study applied a long‐term follow‐up of real‐world data, the results need to be confirmed in larger, prospective, multicenter studies. Although our data indicate a potential benefit of intensive monitoring or follow‐up for patients with CIEDs who have a high risk of developing SAHREs, the absolute benefit of applying this strategy in improving AF detection and reducing stroke needs to be addressed in future studies.

## Conclusions

Individual risk of SAHREs could be stratified using the C_2_HEST score with moderate accuracy and discriminative ability. Patients with CIEDs who have a high risk of developing SAHREs, such as those with C_2_HEST score ≥4, might benefit from a more intensive device examination or continuous remote monitoring, which would need to be tested in prospective studies.

## Sources of Funding

This study was supported by the Chinese Scholarship Council (CSC 201708110232).

## Disclosures

Lip reports consulting for Bayer/Janssen, BMS/Pfizer, Medtronic, Boehringer Ingelheim, Novartis, Verseon, and Daiichi‐Sankyo; and speaking for Bayer, BMS/Pfizer, Medtronic, Boehringer Ingelheim, and Daiichi‐Sankyo. No fees are directly received personally. The remaining authors have no disclosures to report.
